# Aplastic anemia in a patient with papillary thyroid carcinoma complicated with Hashimoto’s thyroiditis: a case report

**DOI:** 10.3389/fonc.2024.1443823

**Published:** 2025-01-07

**Authors:** Fei Wu, Yiwei Xiao, Rui Hai, Xiaodong Chen, Shanshan Liu, Xiangyu Zhou

**Affiliations:** ^1^ Department of Thyroid Surgery, The Affiliated Hospital of Southwest Medical University, Luzhou, Sichuan, China; ^2^ Department of General Medicine, The Affiliated Hospital of Southwest Medical University, Luzhou, Sichuan, China

**Keywords:** papillary thyroid cancer, Hashimoto’s thyroiditis, aplastic anemia, thyroidectomy, hematopoietic function

## Abstract

**Background:**

It is uncommon to come across instances of aplastic anemia in individuals suffering from papillary thyroid carcinoma complicated by Hashimoto’s thyroiditis. Here, a unique case is presented.

**Case presentation:**

A 23-year-old male was admitted to the hospital for “a lump in his right neck”. Laboratory tests revealed a decrease in white blood cells (WBC), red blood cells (RBC), and platelet count (PLT). Bone marrow aspiration revealed an extremely low degree of hyperplasia of hematopoietic cells. Simultaneously, the levels of thyroglobulin antibodies and thyroid peroxidase antibodies were significantly elevated. Neck ultrasound findings revealed nodules in both lobes of the thyroid. Moreover, fine-needle aspiration biopsy indicated the atypical presence of proliferative thyroid epithelial cells. Even after implementing various treatments hematopoietic function could not be restored. However, following a surgery, the patient’s WBC, RBC, and platelet counts gradually returned to normal.

**Conclusions:**

Here, we present a case that thyroid cancer complicated with Hashimoto’s thyroiditis may affect hematopoietic function.

## Introduction

Papillary thyroid carcinoma (PTC) is the most common thyroid malignancy (1). The risk factors for PTC include genetic alterations, radiation exposure, etc (2). Hashimoto’s thyroiditis (HT) (3), an autoimmune disease, is also an independent risk factor for PTC (4). Aplastic anemia (AA) (5) is a form of bone marrow failure, characterized by a hypoplastic bone marrow with profound reduction in hematopoietic stem cells (HSCs), pancytopenia, anemia, infection, and hemorrhage. AA is rarely encountered in patients with PTC complicated with HT. This article reports a case of PTC with HT, with AA considered before admission. After treatment, hematopoietic function is restored, suggesting PTC may affect human hematopoietic function.

## Case report

A 23-year-old male with a persistent right neck mass for more than five months was admitted to our hospital. We found that mass was not accompanied by pain, hoarseness, dysphagia, dyspnea, fatigue, dizziness, palpitations, or other symptoms. However, it could move up and down with swallowing.

### Physical examination

There was no rigidity in the neck, jugular vein ectasia, or abnormal carotid artery pulsation. Hepatic jugular venous reflux was negative. The right neck mass was approximately 1.0 × 1.0 × 1.0 cm^3^ in size, with a hard texture, ill-defined margins, irregular morphology, and no tenderness, but moved while swallowing. No significant enlargement of the cervical lymph nodes was palpated.

### Laboratory tests

Laboratory examination revealed white blood cell (WBC) levels of 2.38 × 10^9^/L, neutrophil levels of 1.19 × 10^9^/L, red blood cell (RBC) levels of 4.20 × 10^12^/L, platelet count of 85 × 10^9^/L, hemoglobin levels of 142 g/L, Free Triiodothyronine (FT3) level of 3.16pg/ml, Free Thyroxine (FT4) level of 1.49ng/dl, Thyroid-Stimulating Hormone (TSH) level of 1.416mU/L, thyroglobulin antibody (TgAb) level of 865.88 IU/mL, thyroid peroxidase antibody (TPOAb) level of 85.82 IU/mL. No obvious abnormalities were seen with respect to the coagulation function, autoantibody profile, anti-neutrophilic cytoplasmic antibody (ANCA), and pre-transfusion tests.

### Imaging studies

**Figure 1:**
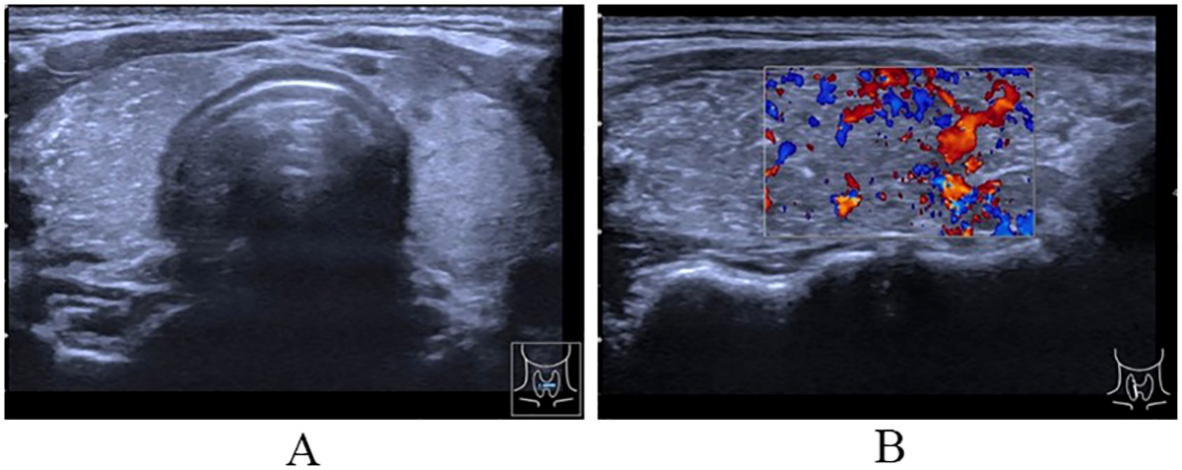
Ultrasound of thyroid. **(A)** The cross-section showing uneven internal echo of the thyroid gland with scattered bright spots; **(B)** The longitudinal section showing diffuse sclerosis of the thyroid gland and abundant blood flow signal in thyroid parenchyma.

The ultrasound analysis of the thyroid and neck lymph nodes reveals that the left thyroid lobe has a superior-inferior diameter of approximately 4.1 cm, a left-right diameter of approximately 1.7 cm, and an anterior-posterior diameter of approximately 1.4 cm. The isthmus has an anterior-posterior diameter of approximately 0.33 cm. The right thyroid lobe has a superior-inferior diameter of approximately 4.3 cm, a left-right diameter of approximately 1.8 cm, and an anterior-posterior diameter of approximately 1.8 cm. The capsule is smooth, and the internal echo is heterogeneous with scattered small bright spots observed within. Multiple hypoechoic lesions are detected in the right neck regions II, III, IV, and the anterior cervical region. These lesions have irregular shapes and clear boundaries, and some of them contain small bright spots. Moreover, no normal lymph hilum is visualized. The largest lesion measures approximately 1.9×0.7 cm^2^. In the left neck regions II and III, multiple hypoechoic lesions are also found. These lesions have regular shapes and clear boundaries, and the largest one among them measures approximately 2.6×0.5 cm^2^. Diagnostic findings: The thyroid shows heterogeneous echo with fine calcifications and rich blood supply, which is categorized as Chinese -Thyroid Imaging Reporting and Data System (C-TRIADS) 4C (**Figure 1**). Abnormal lymph nodes are present in the right neck and anterior cervical region, and the sonographic features of lymph nodes can be observed in the left neck. Enhanced computerized tomography of the neck showed very high-density opacities on the right side of the thyroid gland in the venous phase. Multiple lymph nodes in the bilateral submandibular, carotid artery periosteal, and bilateral posterior cervical space were seen, with the right side as the focus. Laryngoscopy showed chronic pharyngitis.

### Fine-needle biopsy

We performed fine-needle aspiration biopsy twice. The results of the first biopsy were as follows: 1) In the right thyroid gland, very few thyroid epithelial cells and scattered lymphocytes were observed under the microscope (Bethesda I). 2) In the right cervical lymph node, atypical hyperplasia of thyroid epithelial cells and a small number of lymphocytes were observed under the microscope, and metastatic papillary thyroid carcinoma (PTC) could not be excluded. The result of the second biopsy was that in the right thyroid gland, a relatively large number of adenomatous hyperplastic glandular epithelial cells and lymphocytes were present (Bethesda II).

### Diagnosis and treatment

**Figure 2:**
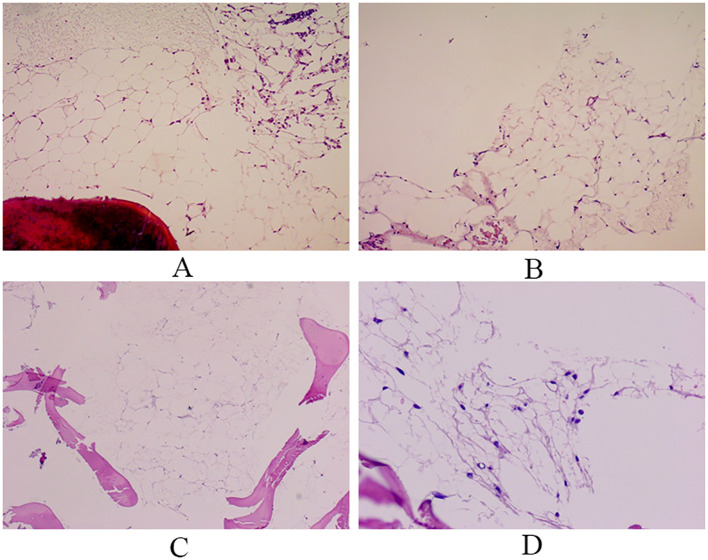
Bone marrow biopsy. Hematopoietic tissue is almost entirely replaced by adipose tissue, and only a few granulocytes are observed. Reticular fiber staining: no reticular fiber hyperplasia observed. (**A**, **B**: Right bone marrow; **C**, **D**: Left bone marrow).

**Figure 3:**
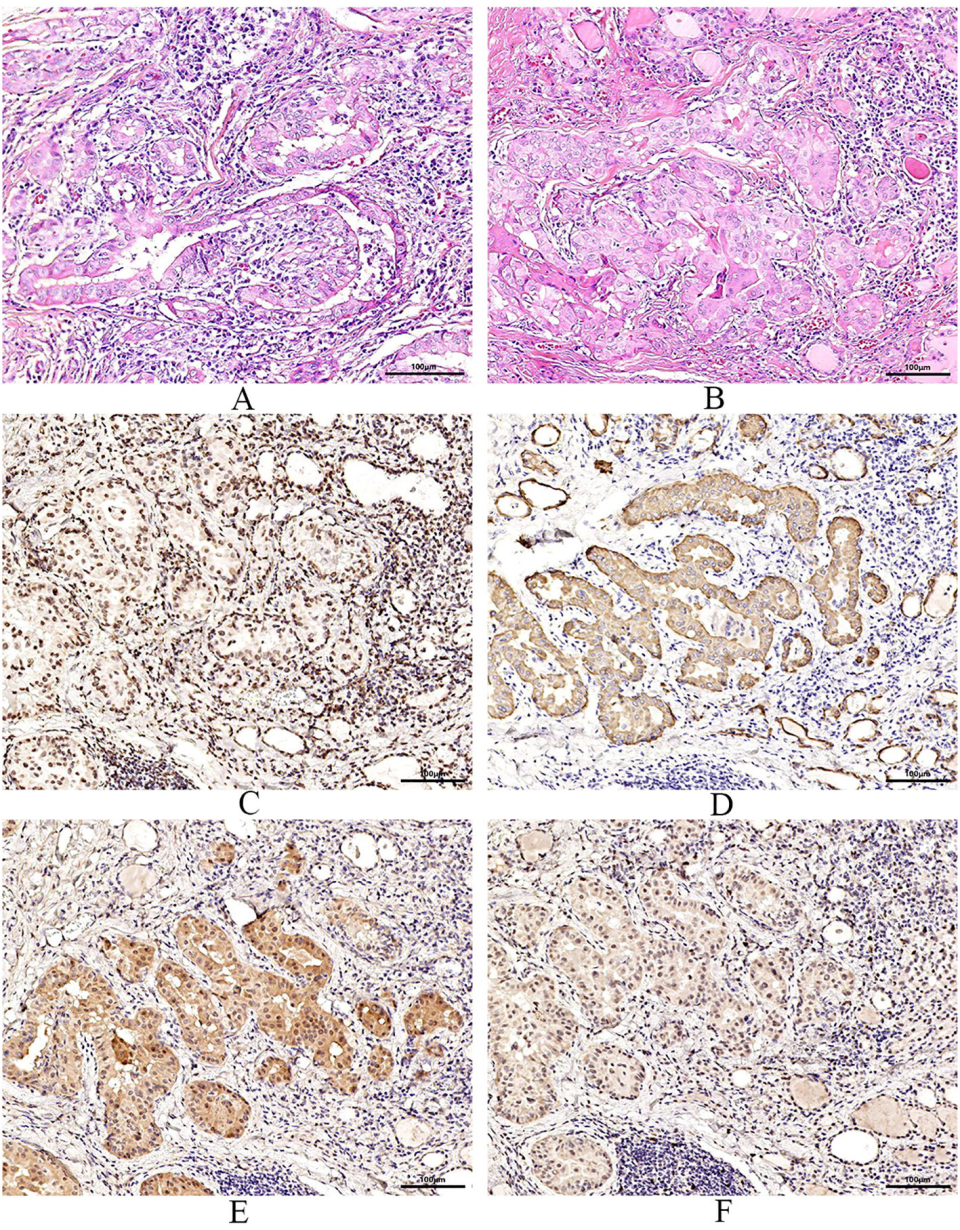
Pathological examination and immunohistochemistry. **(A)** are pictures of the left thyroid gland, **(B)** are images of the right thyroid gland. Pathological features reveal bilateral papillary thyroid carcinoma with Hashimoto’s thyroiditis. Immunohistochemical results of the right lobe of thyroid gland, among, TTF-1 (+) **(C)**, CK-19 (+) **(D)**, Galectin-3 (+) **(E)**, Ki-67 (+, 30%) **(F)**.

**Figure 4:**
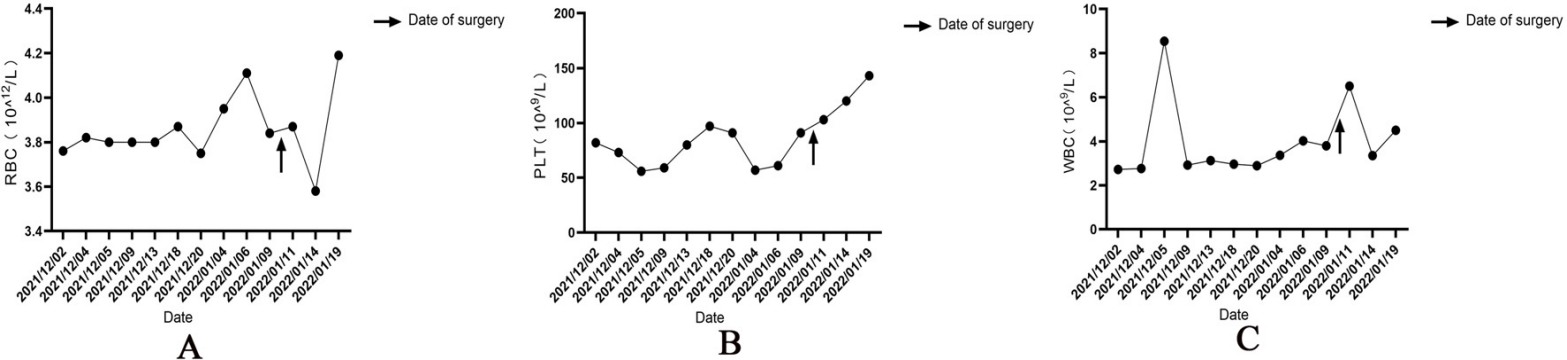
Changes in red blood cell **(A)** and platelet counts **(B)** and white blood cell levels **(C)** before and after thyroid surgery. ’↑’ is the time of operation.

After the patient was admitted to the hospital, the relevant auxiliary examination was completed, and the preliminary diagnosis was as follows: 1) Right side neck mass: thyroid cancer cannot be excluded; 2) Cause of leukopenia remains to be investigated; 3) Cause of thrombocytopenia remains to be investigated; 4) Mild anemia; and 5) Chronic pharyngitis. Although the patient’s thyroid nodules initially led to the consideration of PTC, and surgery was indicated, the patient’s blood routine examination suggested pancytopenia, which was a considerable risk for surgery. We performed bone marrow aspiration, Epstein–Barr virus PCR, human cytomegalovirus quantitative PCR, autoantibody spectrum, evaluation for anemia, and other tests, which showed no obvious abnormality was observed. Bone marrow biopsy implied that hematopoietic cell proliferation was extremely low, and only a few granulocytes and nucleated red cells were seen scattered; megakaryocytes were not seen, and nucleated cells accounted for less than 1% of the bone marrow cavity. No reticular fibrous hyperplasia was observed (MF-0) (**Figure 2**). Blood routine examination showed WBC levels of 2.89 × 10^9^/L, neutrophil levels of 1.30 × 10^9^/L, RBC levels of 3.75 × 10^12^/L, platelet count of 91 × 10^9^/L, hemoglobin level of 128 g/L, and reticulocyte rate (RETIC%) of 1.95%. Other laboratory tests revealed no obvious abnormalities. After consulting with the Hematology Department, we reflected that the patient may have AA. Accordingly, we revised the diagnosis as follows: 1) right neck mass: thyroid cancer cannot be excluded; 2) Suspicion of AA; 3) acute hematopoietic function inhibition; and 4) chronic pharyngitis. We treated the patient with Diyushengbai Tablets (composed of sanguisorbae Radix saponin), compound Zaofan pills (composed of ferrous sulfate, walnut kernels, jujubes, cinnamon, seahorses, and American ginseng), and Leucogen Tablets (2-thiazolidine-4-carboxylic acid). Meanwhile, we also administered intramuscular injection of recombinant human granulocyte colony-stimulating factor (rhG-CSF) and performed platelet transfusion. During treatment, we repeated blood routine examinations several times, but the levels of WBC and RBC and platelet counts were always lower than normal. As the patient had a surgical indication to remove the thyroid, we decided to treat the tumor first. After the preoperative preparation was completed, a total thyroidectomy, central compartment lymph node dissection, and lateral neck lymph node dissection were performed under general anesthesia on January 10, 2022, with intraoperative blood loss of approximately 100 mL. For the specimen obtained from the resection of the left thyroid lobe, it was diagnosed as PTC, with a tumor size of 0.6 × 0.5 × 0.5 cm^3^. There was no vascular infiltration, no intrathoracic dissemination within the thyroid, no thyroid capsule invasion, and no extrathyroidal capsule invasion. The remaining thyroid tissue exhibited changes of Hashimoto’s thyroiditis. Regarding the specimen obtained from the resection of the right thyroid lobe, it was diagnosed as PTC, with a maximum diameter of approximately 0.4 cm. There was no thyroid capsule invasion, no nerve invasion, and no vascular invasion. The surrounding thyroid tissue showed changes of Hashimoto’s thyroiditis. As for the lymph node metastasis situation, cancer metastasis was identified. Specifically, in the submitted “central region”, the metastasis rate was 5/10; in the “left lateral neck region”, it was 0/8; and in the “right lateral neck region”, it was 8/14. Immunohistochemical results of the right lobe of thyroid gland, among, TTF-1 (+), CK-19 (+), Galectin-3 (+), Ki-67 (+, 30%) (**Figure 3**). On post-operative Day-1, we reviewed the blood routine test results of the patient: WBC levels, 6.5 × 10^9^/L, neutrophil levels, 5.58 × 10^9^/L, RBC levels, 3.87 × 10^12^/L, platelet count, 103 × 10^9^/L, and hemoglobin levels, 126 g/L. Multiple blood routine examinations after surgery showed an increase in RBC and WBC levels and platelet count (**Figure 4**), compared with those before surgery. The patient was re-examined on post-operative Day-9, for blood routine tests, and the following was observed: WBC levels, 4.5 × 10^9^/L, neutrophil levels, 2.4 × 10^9^/L, RBC levels, 4.19 × 10^12^/L, platelet count, 143 × 10^9^/L, and hemoglobin level, 141 g/L. Subsequently, the patient recovered and was discharged from the hospital, and also received I131 treatment. After that, we conducted a long-term follow-up on the patient’s thyroid function and blood routine. The results of the thyroid function follow-up on May 7, 2022 were as follows: The FT3 level was 2.62 pg/ml, the FT4 level was 1.60 ng/dl, the TSH level was 1.945 mU/L, the TgAb level was 178.09 IU/mL, the TPOAb level was 33.11 IU/mL, and the thyroglobulin level was <0.04 ng/ml. We continue following up the patient, who was last reviewed in August 2024. The patient’s blood routine results were not significantly abnormal (WBC levels, 5.75 × 10^9^/L, RBC levels, 4.56 × 10^12^/L, platelet count, 125 × 10^9^/L).

## Discussion

Some scholars believed that HT was associated with malignant thyroid tumors as early as in 1955 (6), but the mechanism of PTC complicated with HT remains unclear, and there are various theories, such as endocrine and autoimmune theories. Studies have shown (7) that PTC patients with HT have a better prognosis than PTC patients without HT. Further, AA is a condition of bone marrow hematopoietic failure. Current studies believe that the incidence of AA is associated with the abnormalities in hematopoietic stem cells, microenvironment, and immune function (8). The treatment of AA includes immunosuppressive therapy, androgen therapy, and hematopoietic stem cell inhibition, among others. The chances of spontaneous remission in AA are very rare, in China as well as abroad.

The patient reported in this case study is a young male who was previously healthy, with no anemia-related symptoms. After admission, the patient’s blood routine suggested pancytopenia. We suspected that the patient had AA, which needed to be differentiated from hypoplastic myelodysplastic syndromes (hMDS). hMDS shares morphological and clinical features of both MDS (dysplasia, genetic lesions, and cytopenias) and AA (hypocellularity and autoimmunity) (9). A diagnosis of hMDS is still a matter of debate, as it lacks a clear place in the recent WHO classification. The main distinguishing point between the two is dysplasia. Other morphological features that support a diagnosis of hMDS include excess bone marrow blasts (≥2%), ring sideroblasts, extensive fibrosis, and circulating pseudo-Pelger-Huet cells (10). Thus, following consultation and bone marrow biopsy, we suspected that the patient was suffering from AA. However, considering the patient’s strong preference for having thyroid surgery first, we refrained from applying the standard immunosuppressive therapy. Since the patient had requested the operation, we decided to correct the anemia first, so that the patient could tolerate the surgery, after which, we treated the hematopoietic dysfunction. Diyushengbai Tablets contain sanguisorbae Radix saponin, which has been demonstrated to promote hematopoiesis in bone marrow (11). Zaofan Pills, composed of ferrous sulfate, walnut kernels, jujubes, cinnamon, seahorses, and American ginseng, can be utilized for the treatment of aplastic anemia, neutropenia, thrombocytopenia, myeloproliferative disorders, and bone marrow injury induced by radiotherapy and chemotherapy (12). Both Diyushengbai Tablets and Zaofan Pills are two widely employed traditional Chinese patent medicines in China. We anticipate that the combined application of these two traditional Chinese patent medicines along with Leucogen Tablets and rhG-CSF can ameliorate the anemia symptoms of patients. Surprisingly, even after administering several treatments to try to correct the anemia before the surgery, none worked. However, after surgery, all blood cell counts were recovered.

Both hyper and hypothyroidism can cause anemia, the reason may be increased food intake, malnutrition, iron utilization disorders, inhibition of hematopoietic function, or malabsorption of vitamin B12 (13). In addition, cases wherein drugs used to treat hyperthyroidism have led to AA have been reported in China and abroad (14). Reports have indicated that patients with AA develop abnormal autoimmunity against hematopoietic stem cells, leading to severe suppression of these cells in some cases (15). And the effectiveness of immunosuppressive therapy in treating these patients confirms the presence of immune dysregulation within their bodies. Hashimoto’s thyroiditis is an autoimmune disease characterized by the presence of high titers of thyroid-related antibodies in the patient’s blood and it may be accompanied by other autoimmune diseases (16). Therefore, we speculate that HT and AA share a common autoimmune mechanism in the pathogenesis. However, patients with PTC complicated with AA, are exceedingly rare (17), and it is difficult to determine whether there is a link between the two. Anemia is a common complication in malignant tumors. Cancer cells infiltrating the bone marrow, blood loss caused by tumors, lack of hematopoietic raw materials caused by tumors, release of tumor-related cytokines, and bone marrow suppression caused by chemotherapy, are all causes of cancer-related anemia (18). In this case, the patient was diagnosed with PTC and HT, after admission and postoperative pathological analysis. Meanwhile, the patient presented with pancytopenia, and was possibly suffering from AA. No tumor infiltration was found in the patient’s bone marrow puncture biopsy, and no deficiency of iron and B vitamins was found in anemia-related laboratory tests. However, after surgery was performed, the patient’s hematopoietic function gradually recovered. There are very few articles on the relationship between thyroid cancer and aplastic anemia. Only one Japanese scholar has proposed that there is an underlying autoimmune background for the simultaneous occurrence of aplastic anemia and Hashimoto’s thyroiditis complicated by thyroid carcinoma (19). We speculated that PTC could have produced some cytokines affecting the hematopoietic function of the bone marrow, and even inducing AA. However, the specific correlation between PTC and AA needs to be studied further.

## Data Availability

The original contributions presented in the study are included in the article/supplementary material. Further inquiries can be directed to the corresponding authors.
